# Eating Disorders Risk Assessment and Body Esteem among Amateur and Professional Football Players

**DOI:** 10.3390/nu16070945

**Published:** 2024-03-25

**Authors:** Wiktoria Staśkiewicz-Bartecka, Marek Kardas

**Affiliations:** Department of Food Technology and Quality Evaluation, Department of Dietetics, Faculty of Public Health in Bytom, Medical University of Silesia in Katowice, ul. Jordana 19, 41-808 Zabrze, Poland; mkardas@sum.edu.pl

**Keywords:** eating disorders, body esteem, football players, body perception, football professional, football amateur

## Abstract

Background: The purpose of this study was to assess the risk of eating disorders and attitudes toward one’s own body among football players at amateur and professional levels. Methods: The study included 90 players from football clubs located in the Upper Silesia Metropolitan Area, participating in the 4th and 5th leagues in Poland. A survey questionnaire was used to conduct the study, which consisted of a metric section, an Eating Attitudes Test, and Body Esteem Scale questionnaires. The players were divided into two groups according to their sports level. Results: Results showed that 24.4% of players were overweight, while 75.6% had a normative body weight. Approximately 16.7% met the criteria indicating susceptibility to an eating disorder. Body Esteem Scale interpretations revealed moderate body appraisal among players. Conclusions: Both amateur and professional athletes showed no significant difference in eating disorder risk, but professionals rated their bodies higher. Social media use, particularly on Twitter and Instagram, is correlated with eating disorders, with longer daily use associated with lower body ratings.

## 1. Introduction

Nowadays, sports culture has a huge impact on self-perception and eating behavior [1]. Among various sports, football is gaining popularity at the amateur and professional levels, attracting both youth and adults to physical activity on the field [2]. As interest in the sport grows, it is important to understand the potential risks associated with the physical well-being and mental health of football players. There is a growing awareness that football players may be a group at increased risk for eating disorders (ED) and negative perceptions of their bodies [3,4]. Dissatisfaction with one’s physical appearance, along with the desire to be thinner to improve athletic performance, can lead to the development of ED. Individuals who are dissatisfied with their bodies may engage in unhealthy behaviors, such as restricting food intake and using ergogenic or laxative drugs, which can ultimately contribute to the development of ED [5,6]. Athletes are a group that is particularly vulnerable to their occurrence. A review of the literature shows that ED is more common in athletes than in the general population [5]. Such a phenomenon is particularly noted in sports focusing on thinness and low body mass, such as athletics, figure skating, and gymnastics [1].

The incidence of eating disorders is influenced by socio-cultural factors; athletes, due to generally accepted norms and society’s notion of the appearance of a physically active person, are under constant pressure [5]. In addition, they often derive nutrition knowledge from incorrect sources or unqualified individuals. The pursuit of satisfaction and perfection can lead to extreme body mass control methods and dietary restrictions [6].

EDs have been classified according to the criteria in the two major mental illness classification systems. European countries use the ICD-11 (International Classification of Diseases and Related Health Problems). In the United States, the DSM-5 (Diagnostic and Statistical Manual of Mental Disorders), developed by the APA (American Psychiatric Association), is used. Both classifications provide detailed descriptions of different types of ED, such as anorexia nervosa (AN), bulimia nervosa (BN), and other related disorders, allowing professionals to accurately diagnose and plan appropriate treatment for those affected [7,8].

AN is characterized by restriction of food intake, leading to significant malnutrition and weight loss. BN is manifested by cycles of overeating followed by compensatory behaviors such as vomiting or excessive exercise. Paroxysmal overeating (PO) is associated with recurrent bouts of overeating, which can lead to excessive weight gain. Restrictive EDs are characterized by a reduction in the amount of food consumed but do not meet all the criteria for anorexia nervosa [8,9,10].

The DSM-5 and ICD-11 classifications provide an important diagnostic tool for mental health professionals to accurately diagnose and plan appropriate treatment for those suffering from ED. It also points to the need for further research into the mechanisms of onset and treatment of these disorders to provide effective help for those affected [8,11].

Research increasingly confirms the significant influence of the media on the development of ED and negative perceptions of one’s own body among the general population as well as athletes [12,13,14]. Media messages often promote unattainable standards of beauty and body, which can lead to unreasonable expectations about appearance and lead to negative effects on self-esteem. In the case of football players, who are often subjected to intense pressure to maintain a slim and fit physique, the media can further intensify the pressure to maintain an ideal physical appearance [15,16]. Studies show that comparison with ideals of appearance communicated through social media results in increased levels of dissatisfaction with one’s own body. Such a phenomenon consequently leads to risk factors for ED [16]. Moreover, this problem affects both young people and adults. The above behavior carries very serious consequences, contributing to the development of depression and dietary restrictions, and thus negative health consequences [17].

Football players may be dissatisfied with their bodies for a variety of reasons, including social pressures, expectations of athletic performance, or comparisons to other players [4]. Social media often promote idealized standards of beauty and physique, which can lead to a relentless pursuit of physical perfection [3]. Dissatisfaction with one’s body can cause stress and lower self-esteem, which in turn can lead to dietary restrictions, excessive physical activity, or the use of compensatory measures. Long-term stress related to body dissatisfaction and constant pressure to meet ideal physical standards can increase the risk of ED among football players [3].

The present study aims to assess the risk of ED and the perception of one’s own body among football players at the amateur and professional levels. The research hypothesis is that football players have an ED risk and a negative evaluation of their bodies. It is also predicted that there will be differences in self-perception and ED risk between amateur-level football players and those at the professional level, with professional-level players likely to exhibit higher risk and more critical self-perceptions of their bodies due to greater environmental pressures and professional expectations.

## 2. Materials and Methods

### 2.1. Procedure for the Study

The survey was conducted from December 2023 to February 2024. The survey included players of football clubs located in the Upper Silesia Metropolitan Area. The method used to obtain the results was CAWI (Computer-Assisted Web Interview); the survey was conducted using a web form, which is an acceptable method in psychological research. The Google Forms platform was used for data collection due to its ease of use, accessibility, and ability to customize the questionnaire for the study. The questionnaire was made available to participants by sending a link to the survey to players of the clubs included in the study. 

Dedicated sampling was used in the study. With this method, the sample was selected to represent characteristics, specific experiences, and traits related to the topic of the study. Determination of precise selection criteria, such as the location of the clubs (Upper Silesian Metropolis), playing level (4th and 5th level of competition in Poland), and gender are key features to achieve the objectives of the study.

All study participants were informed about the purpose of the study and its anonymity and were asked to accept the rules for sharing data. Information about informed and voluntary participation in the study was at the beginning of the questionnaire. The World Medical Association’s Declaration of Helsinki guided the conduct of this study. The study was approved by the Bioethics Committee of the Silesian Medical University in Katowice (BNW/NWN/0043-3/641/35/23, date of approval: 22 September 2023) in light of the Law of 5 December 1996, on the Profession of Physician and Dentist (Journal of Laws 2016, item 727).

The sample size was estimated based on the formula for minimum sample size, and data substituted from the formula took into account the total number of football players at a given level of play in clubs located in the Upper Silesia Metropolis. This ensured that a representative group of study participants was obtained. The study used the following formula:

$$N_{min}=\frac{N_{P}(\alpha^{2}\cdot f\left(1-f\right))}{N_{P}\cdot e^{2}+\alpha^{2}\cdot f(1-f)}$$

where: *N*_*min*_—minimum sample size, *N*_*P*_—population size, α—confidence level, *f*—fraction size, *e*—assumed maximum error.

### 2.2. Participants

The survey included 98 players who are players of football clubs located in the Upper Silesia Metropolitan Area. The response rate was 85.71%. The form was filled out correctly by 90 football players aged 18–40. The players belonged to clubs participating in the 4th and 5th league levels of men’s football in Poland. The survey was conducted during the break between the autumn and spring rounds of the 2023/2024 league season; all players were surveyed during the transition period to obtain comparable results. Table 1 shows the football league system in Poland concerning the level of competition.

Players playing at the 5th league level were classified as amateur footballers (AF), while those playing at the 4th league level were a group of professional footballers (PF) according to the statutes of the Polish Football Association (PZPN) [18].

The criteria for inclusion in the study group were the following conditions: (1) voluntary participation in the study and complete completion of the questionnaire, (2) age 18 or older, and (3) status as an active club player during the study.

### 2.3. Survey Tools

A survey questionnaire was used to conduct the study, which consisted of a metrics section (respondent’s data: age, height, body mass, chronic diseases (including mental health problems such as depression, eating disorders, neurosis, etc.) and medications taken, education, field position, sports seniority, number of training sessions per week, sources of nutritional knowledge, exclusions of food products, information on social media use, and Eating Attitudes Test (EAT-26) and Body Esteem Scale (BES) questionnaires.

### 2.4. Body Mass Index (BMI)

The nutritional status of the participants was assessed by body mass index, calculated according to the formula:

$$BMI=\frac{body\;weight\;(kg)}{{height\;(m)}^{2}}$$

The results were interpreted according to the World Health Organization (WHO) guidelines, as shown in Table 2.

### 2.5. EAT-26

The study used a screening tool for assessing ED risk, the American Eating Attitudes Test developed by Garner et al. [20]. This questionnaire is a standardized tool for identifying ED risk symptoms. It was developed to screen both those with a clinical diagnosis and those at risk for AN, BN, or obesity. EAT-26 is one of the most widely used diagnostic tools in eating disorder prevalence studies worldwide. The author of the Polish standardization of the tool is K. Włodarczyk-Bisaga [21]. The interpretation of the EAT-26 questionnaire consists of three “referral criteria” that determine whether the respondent should report for further evaluation of ED risk:

1. The final score on the EAT-26 questionnaire is the sum of the scores obtained from 26 questions on attitudes toward nutrition. Questions 1 through 25 are scored as follows: Always = 3 points; Usually = 2 points; Often = 1 point; Other answers = 0 points. Question 26, meanwhile, is scored oppositely: Never = 3 points, etc. The total score of the test can range from 0 to 78. A person scoring ≥20 is considered at risk of developing an ED and should consult a specialist for further diagnosis.
2. Questions about behavioral patterns may suggest the presence of symptoms of an ED or recent significant weight loss. These questions focus on compensatory behaviors such as the use of laxatives, provoking vomiting, overeating, excessive physical activity, and rapid and significant weight loss in a short period. An affirmative answer to any of these questions may suggest the presence of abnormalities and the need for further diagnosis of ED.
3. The survey includes precise questions about respondents’ height, weight, and gender, which are used to calculate body mass index. BMI can suggest possible risks of ED if weight is low compared with age standards. Evaluating BMI in the context of respondents’ height, weight, and gender data identifies potential risks and the need for further analysis of subjects. Table 3 shows interpretations of BMI compared with age standards.

### 2.6. BES

The subjects’ body image was measured using The Body Esteem Scale [22]. BES, in a Polish adaptation by Lipowska and Lipowski [23], allows one to assess one’s attitude toward one’s own body. The scale contains 35 items in three subscales, different for men and women. The subscales for men are physical attractiveness, upper body strength, and physical condition. Responses are given on a five-point Likert scale, where “1” means strong negative feelings, “5” means strong positive feelings, and “3” is neutral [22,23]. 

The physical attractiveness subscale for men is based on an assessment of the features that mainly determine the description of a man as handsome. It includes both facial features and body parts such as hips and feet. Although the evaluation of sexual organs affects the score on this scale, it does not take into account their function or sexual activity. The body strength subscale combines assessments of different parts of the body (such as the arms or chest), as well as their function and fitness, determining strength and activity. Physical fitness refers to assessments of body strength and agility [23].

A table of norms for men including age categories and stenes was used to interpret the BES results. The table is divided into 6 age groups and 10 stens. If the value of the collected points fluctuated on the sten scale between 1–3—the body assessment was low, 4–7—medium, and 8–10—high [23]. Sten scores (short for “Standard Ten”) are standardised 1–10 scores commonly used in psychometric testing. Shown in Table 4.

### 2.7. Statistical Analysis

Statistical analyses were performed using Statistica v.13.3 (Stat Soft Poland, Kraków, Poland) and the R package v. 4.0.0 (2020) under the GNU GPL (The R Foundation for Statistical Computing). To present quantitative data, mean values and standard deviations (X ± S) were calculated; for qualitative data, percentage notation was used. 

Compliance with the normal distribution was checked using the Shapiro–Wolf test. The significance of differences between amateur and professional football players was assessed using Student’s t-test for two parametric groups, analysis of variance (ANOVA) for three or more parametric groups, Mann–Whitney U-test for two non-parametric groups, and Kruskal–Wallis test for three or more non-parametric groups.

To examine the relationship between EAT-26 and BES scores, Cramer’s V coefficient was used. The value of this coefficient makes it possible to measure the strength of the relationship between two categorical variables, in this case, the ED development risk score and the body score.

A value of *p* < 0.05 was used as a criterion for statistical significance.

## 3. Results

### 3.1. Sample Characteristics

Ninety football players participated in the study after taking into account the inclusion and exclusion criteria. The players were divided into two groups according to their sports level; the first group (n = 59) consisted of amateur footballers (AF), while the second group (n = 31) consisted of professional footballers (PF). The participants in the study had secondary (n = 52, 57.78%) or higher (n = 38, 42.22%) education. Six of the football players had chronic diseases (3-hypothyroidism, 2-stomach mucositis, 1-hypertension); only hypothyroid patients were taking permanent medications (Letrox or Euthyrox). The characteristics of the study group are shown in Table 5.

The main source of nutritional knowledge for all players was the Internet (n = 40; 44.44%), followed by a nutritionist (n = 18; 20%), with a similar percentage of players indicating a coach (n = 11; 12.22%), other players (n = 10; 11.11%) and friends (n = 9; 10%). Football players were indicated least often by research (n = 2; 2.22%), and there was no correlation between sports level and source of nutritional knowledge (*p* = 0.071).

The majority of respondents did not exclude any group of food products from their diet (n = 58, 64.44%). Football players in both groups were most likely to exclude products containing lactose (AF = 25.64%, PF = 19.35%), and there was no relationship between sports level and exclusion of food groups from the diet (*p* = 0.579). The athletes were also asked how they adapted their nutrition to physical activity. A statistical relationship was found between sports level and dietary changes related to physical activity. Professional football players are more likely to increase their carbohydrate and protein intake and increase their energy intake on training or match days, while amateurs are more likely to limit their intake of sweets (Table 6).

The athletes were asked questions about their social media activity and about comparing their physique to other athletes. A statistically significant relationship was found between time spent using social media and comparing their body image to that of other athletes. Detailed information is shown in Table 7.

### 3.2. BMI of Participants

**Figure 1:**
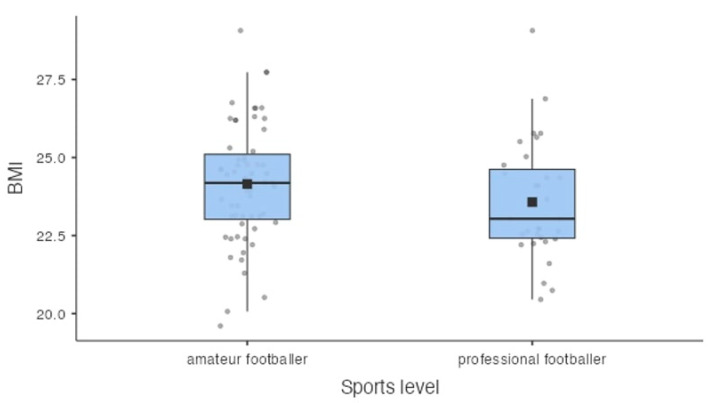
Distribution of BMI values in a group of amateur footballers (n = 59) and professional footballers (n = 31). Note: The black square indicates the average value.

Based on the calculated BMI of the players, it was shown that 24.4% (n = 22) of the respondents were overweight, normative weight was 75.6% (n = 68) of the football players, and no player was underweight or obese. Among amateur football players, 25.4% (n = 15) were overweight, while normal body weight was 74.6% (n = 44) of respondents. Analyzing the BMI values among professional athletes, it was found that 22.6% (n = 7) of them were overweight, and 77.4% (n = 24) had a normative body weight. Detailed information is shown in Figure 1. Based on the respondents’ calculated BMI and subsequent comparison with age norms, it was found that 3.4% of amateur and 3.2% of professional football players were underweight compared with age norms. There was no significant effect of sports level on low body weight compared with age norms (*p* = 0.967).

### 3.3. Risk of ED

Based on the EAT-26, Part A questionnaire score, it was estimated that 8.9% of respondents (amateurs and professionals) were at risk for ED and should see a specialist for further diagnosis. No significant differences were found between sports level and total EAT-26, Part A test score, which can indicate the risk of developing an ED (*p* = 0.171). However, statistically significant differences were found between nutritional status interpreted through the BMI value according to WHO recommendations and the total score of EAT-26, Part A test (*p* = 0.009). Overweight athletes were more likely to have an increased risk of ED. No such relationship was shown when analyzing interpretations of BMI versus age norms (*p* = 0.582).

According to the accepted results in the behavioral questions from the EAT-26 test, Part B, it was estimated that 10.2% of amateur and 9.7% of professional football players met the criterion that may indicate a risk of developing an ED. There was no significant effect of sports level on EAT-26 test scores for behavioral questions (*p* = 0.942). However, statistically significant differences were found between nutritional status interpreted through BMI values according to WHO recommendations and the total EAT-26 Part B test score (*p* = 0.001). Overweight athletes were more likely to have an increased risk of ED. No such difference was shown when analyzing interpretations of BMI compared with age norms (*p* = 0.577).

**Figure 2:**
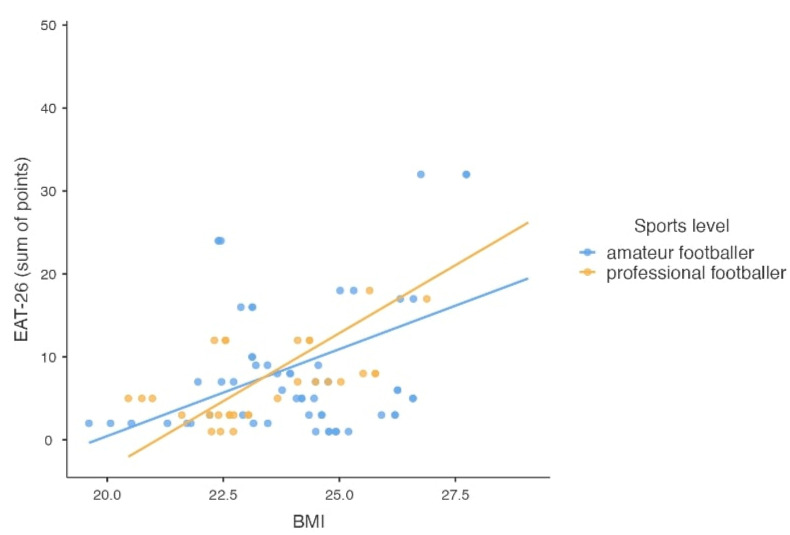
The scatter plot of the total score obtained in the EAT-26 test and the value of BMI, taking into account the athletic level of the subjects (n = 90).

Based on the overall results and interpretation of the EAT-26 questionnaire, it was found that 16.7% of respondents (both amateurs and professionals) met at least one of three criteria that may indicate the likely existence or susceptibility to an ED. These individuals should see a specialist for further diagnosis. There was no significant effect of sports level on the overall EAT-26 score (*p* = 0.487). Statistically significant differences were found between nutritional status interpreted through BMI values according to WHO recommendations and the total EAT-26 test score (*p* = 0.004). Overweight athletes were more likely to have an increased risk of ED. The score obtained in the EAT-26 test about BMI values is shown in Figure 2.

Significant differences were also found when analyzing interpretations of BMI compared with age norms versus the total EAT-26 test score (*p* = 0.001). Athletes with low body weight compared with age standards were more likely to be prone to ED. There was a correlation between the type of social media most frequently used and the total EAT-26 test score (*p* = 0.026). Twitter and Instagram users were more likely to have or be susceptible to an ED. Time spent using social media was not a significant determinant (*p* = 0.268) (Table 8).

### 3.4. Attitude towards One’s Own Body

According to the BES interpretation, football players were characterized by moderate body ratings in the categories of physical attractiveness, upper body strength, and physical fitness. In addition, significant differences were observed between the groups of amateur and professional athletes. When analyzing the mean value according to the sten scale, professional athletes had higher mean sten values in all analyzed subscales (*p* = 0.008; *p* = 0.031; *p* = 0.039). Similarly, professional football players were characterized by higher body scores in all subscales as interpreted for BES (*p* = 0.016; *p* = 0.037; *p* = 0.004). Detailed results are shown in Table 9.

**Figure 3:**
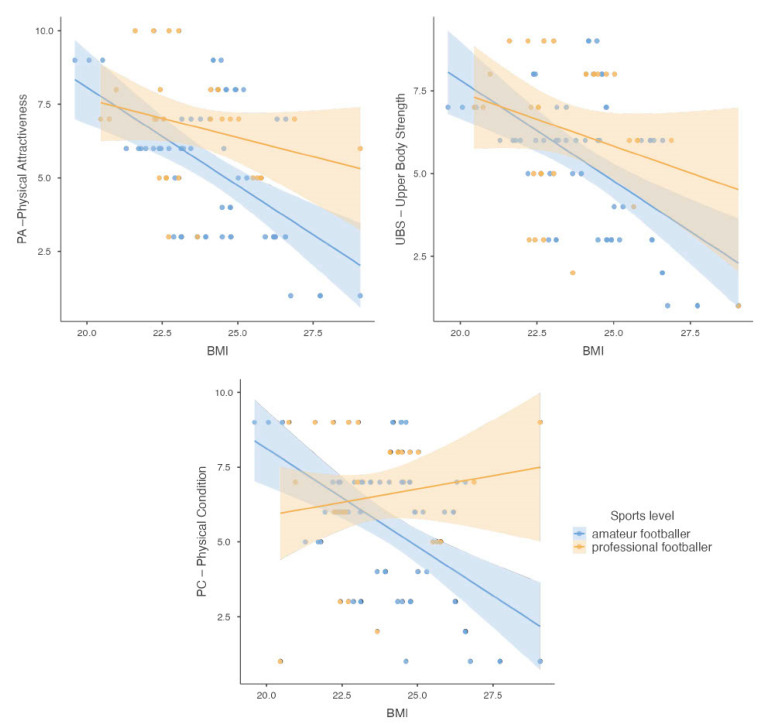
Scatter plots showing the sum of scores obtained in the BES for the subscales: PA (Physical Attractiveness), UBS (Upper Body Strength), and PF (Physical Fitness), according to the value of the BMI index, taking into account the participants’ sports level.

The difference between groups of athletes according to the interpretation of BMI and self-body attractiveness assessment was also analyzed. It was shown that athletes with normal body weight had higher satisfaction on the self-assessment subscales of physical attractiveness (*p* = 0.002) and upper body strength (*p* = 0.008) compared with overweight athletes. In contrast, there was no relationship between the interpretation of BMI and the physical fitness subscale (*p* = 0.087). The sum of the BES scores for all three subscales according to BMI values is shown in Figure 3.

In addition, differences between preferred social media and time of use and BES interpretation were also analyzed. While no statistically significant difference was found in the physical attractiveness and upper body strength subscales (*p* = 0.091; *p* = 0.446), Twitter users were shown to have higher self-esteem on the physical fitness subscale, while Instagram users had lower self-esteem (*p* = 0.002). Analyzing the time claimed on social media use showed differences between athletes. Athletes using social media more than 3 h a day were characterized by lower body ratings in all three subscales (*p* = 0.001; *p* = 0.003; *p* = 0.001).

The relationship between the interpretation of the EAT-26 test score and the interpretation of BES scores on all three subscales was also analyzed. A statistically significant moderate association was found between the risk of developing ED and scores on the subscales of body strength (0.014) and physical fitness of football players (*p* = 0.005). There was no significant association between the physical attractiveness subscale and the risk of developing ED (*p* = 0.085). The results are presented in Table 10.

## 4. Discussion

The study was aimed at assessing the risk of ED and attitudes toward one’s own body among football players at different levels of sports—amateur and professional. The results of the study are relevant to both the physical and mental health of the players and have important implications for coaches, sports doctors, and mental health specialists.

Data analysis showed an association between BMI and the risk of ED in football players. According to the results, overweight players showed a higher prevalence of increased risk of ED. This is an important observation, suggesting the need to monitor nutritional health among this group of athletes. The study also observed that normal-weight athletes showed higher feelings of satisfaction with their self-assessment of physical attractiveness and body strength compared with overweight athletes. These results suggest that maintaining a normal body weight may have a beneficial effect on psychosocial aspects and athletes’ well-being. 

However, it should be noted that the interpretation of BMI can be limited, especially for athletes. The BMI score does not account for differences in body proportions, nor does it distinguish between muscle mass and body fat. In the case of football players, who often have increased muscle mass associated with high levels of physical activity, a high BMI may be the result of high muscle mass rather than overweight or obesity [24,25,26].

The results of the analysis of the EAT-26 questionnaire showed that about 8.9% of respondents (both amateur and professional) are at risk of developing an ED and should see a specialist for further diagnosis. Similarly, an analysis of behavioral questions from the EAT-26 test, Part B, showed that about 10.2% of amateur and 9.7% of professional football players may exhibit behaviors that suggest a risk of developing an ED. Again, there was no significant effect of sports level on the EAT-26 test score for behavioral questions. The overall results of the interpretation of the EAT-26 questionnaire showed that about 16.7% of respondents met at least one of the three criteria that may indicate the probable existence or susceptibility to an ED. This indicates the need to consult a specialist for further diagnosis. It is worth noting that no significant differences were observed between sports level and total EAT-26 test score, suggesting that both amateurs and professionals may be at risk of developing an ED.

A study by McDonald et al. [27] aimed to examine gender differences in overall scores on the EAT-26 in National Collegiate Athletic Association (NCAA) athletes participating in “lean” and “non-lean” sports. Using a self-report questionnaire, 121 athletes were surveyed. The results showed a significant effect of sport type on attitudes and eating behavior. Men who played “non-lean” sports scored higher on the attitudinal part, while men who played “lean” sports scored higher on the behavioral part. There was also an interaction between gender and sport type. Female athletes, regardless of sport type, scored similarly on both sections of the EAT-26. The study indicates that athletes, regardless of gender or sport type, may suffer from ED symptoms, and gender differences may be smaller in athlete populations [27].

Prather et al. [28] conducted a study among 220 female football players representing various levels of the sport. The results showed that some of these female athletes experienced menstrual problems and suffered from stress fractures of the lower limb. In addition, a delay in the onset of first menstruation was observed despite a normal BMI and healthy body perception. An analysis of EAT-26 revealed that about 8% of female athletes had scores indicating a risk of ED [28].

A study by de Sousa Forest et al. [29] aimed to develop a socio-sport model of ED in Brazilian male athletes. They studied 321 athletes from 18 different sports, assessing ED using the EAT-26, body fat dissatisfaction using the Body Shape Questionnaire (BSQ), and muscular concerns on the Desire for Muscularity Scale (DMS). The results of the study indicate that q of the EAT-26 scale, 15% of athletes exhibited risk behaviors related to ED (EAT-26 ≥ 20 points) [29]. These results indicate that a higher percentage of men are at risk of developing ED than the results of our study, and athletes in other sports who qualified for the de Sousa Forest et al. study may be at higher risk than football players [29].

A study by Abbot et al. [30] examined the prevalence of ED in elite male and female football players and the impact of perfectionism on this risk. The results showed that male football players had higher scores on the EAT-26 compared with controls, suggesting a higher risk of ED. In contrast, in women, the prevalence of ED risk was higher in the control group than among female players, although there were no differences in EAT-26 scores between female and male football players. Perfectionism was found to be a significant predictor of ED risk in both men and women [30].

A study by Pustivsek et al. [5] aimed to examine risk factors for ED among different groups of professional athletes compared with non-athletes, with a focus on aesthetic sports and ball games. The results showed that the percentage of ED sufferers was significantly higher among athletes involved in aesthetic sports than in the other groups (17% compared with 3% in ball game athletes and 2% in non-athletes).

The study suggests that sport-specific factors, such as athletic pressure and early specialization, may have a significant impact on the risk of ED, especially for aesthetic sports [5].

Results related to self-perceived body image showed significant differences between amateur and professional athlete groups. Professional football players had higher mean scores on the sten scale in all subscales analyzed and higher body evaluation according to classification (BES) in the body strength and fitness subscale. This suggests that professional athletes may experience greater pressure related to physical appearance and expectations for athletic performance.

The study by Bialek et al. [31] assessed body composition, self-perception, and body evaluation using the BES. The study included 150 women: 50 volleyball players, 50 bodybuilding and fitness athletes, and 50 female students who served as the control group. Perceptions of one’s own body in terms of evaluation of individual body parts were highest among bodybuilding and fitness athletes, while volleyball players had the best results in terms of body conditioning. Most female volleyball players were dissatisfied with their current body weight, as were the women in the control group, in contrast to female bodybuilding and fitness athletes, who were most often satisfied with their current body weight [31].

The subject of the study by Kong et al. [32] was the assessment of body dissatisfaction vs. ED symptomatology among 320 elite and recreational athletes participating in sports focused on lean body shape and sports not focused on lean body shape. Athletes in sports focused on a lean physique reported higher levels of body dissatisfaction and greater ED symptomatology, regardless of level of participation. Elite athletes reported higher levels of body dissatisfaction and greater ED symptomatology regardless of sport, and differences between recreational and non-competitive athletes were not found. More than 60% of elite athletes from sports focused on lean body shape, and sports not focused on lean body shape reported pressure from coaches regarding body shape [32].

In addition, the study found that social media use can affect self-perception and the risk of ED among football players. Twitter and Instagram users were more likely to show the likelihood of having or being susceptible to an ED. This suggests that coaches and support staff should be aware of the impact of social media on players’ psychological and emotional well-being, and this may require implementing social media management strategies among players.

A survey of young adults in the U.S. and other studies indicate that there is an association between the use of social media, such as Facebook, and body image concerns and eating problems. Participants reported higher levels of body image concerns when using Facebook, such as feeling embarrassed or motivated to change their appearance after comparing themselves to others’ photos [33]. Additionally, a study involving American college students found that about 10% of female participants reported that photos posted by others on Facebook negatively affected their body image, which can lead to a decrease in body satisfaction resulting from social comparisons [34].

Similar phenomena have also been observed in other countries, such as Kuwait, where more than 20% of female students admitted that they followed diets to lose weight due to their Internet use [35]. These qualitative data confirm the existence of a link between Internet and social media use and body image and dietary problems, an important issue that requires attention and further research. The availability of the Internet and the ubiquity of social media call for a more conscious approach to promoting positive body image and healthy lifestyles among young people, including athletes.

Overall, the study’s findings underscore the need to integrate mental health aspects into the training and management of a football team. Coaches, sports physicians, and mental health professionals should be aware of the risk of ED among players and take appropriate preventive and intervention steps to provide comprehensive care for players. Implementing educational programs and psychological support can be key to promoting healthy eating attitudes and positive body perception among football players at various levels of the sport.

### Strengths and Limitations

The survey conducted among football club players had several strengths. The study used dedicated sampling, which ensured that participants were representative of characteristics related to the study topic. In addition, standardized diagnostic tools such as the EAT-26 and the BES were used, which allowed an accurate assessment of the participants’ risk of ED and self-perception of their bodies. Analyzing the available literature, this is the first study with amateur and professional football players to assess the difference between self-perception and the possible development of ED in this group of athletes.

However, the study also had some weaknesses. The limited representativeness of the sample, which included only football players in one region, may make it impossible to generalize the results to other populations. In addition, the study focused only on men, which limits the ability to analyze potential gender differences in the results. The use of self-assessment tools may introduce some distortion in the results due to the subjective perceptions of respondents. However, appropriate precautions were taken in surveying to ensure participants’ anonymity to reduce public pressure, clearly mark the objectives of the survey, and encourage honesty and openness in their responses. Additionally, the short survey period may limit the analysis of long-term trends and changes. In the continuation of the study, it is also worthwhile to include a body composition analysis instead of BMI, which will allow for the assessment of the proportion of muscle mass and body fat and the relationship of the values to test results. The study should be expanded to include additional components due to the interesting results of this study. Conducting more detailed analyses on specific types of content and interactions on social media platforms will allow us to understand better the mechanisms affecting the correlation between social media use and eating disorders and propose more effective intervention and prevention strategies.

## 5. Conclusions

The results of a study on the risk of ED among football players showed that about 17% of the participants had a risk of ED. Both amateur and professional players did not differ significantly in this risk, but professional athletes rated their bodies higher in all aspects analyzed, i.e., physical attractiveness, upper body strength, and physical fitness.

Analysis of BMI showed that overweight athletes were more likely to have an increased risk of ED. In contrast, normal-weight athletes had higher self-assessments of physical attractiveness and body strength compared with overweight athletes.

The use of social media, especially Twitter and Instagram, was associated with the likelihood of having or being susceptible to an ED. Twitter users showed a higher self-assessment of physical fitness, while Instagram users had a lower self-assessment. Players who used social media for more than three hours a day had lower body ratings.

The results underscore the importance of awareness and education about eating disorders among athletes and the need to pay attention to factors related to body evaluation and social media use in the context of athletes’ mental health. The study’s findings may increase athletes’ awareness of the risk of ED among athletes, which may lead to earlier recognition and intervention for eating problems. Training staff need to provide education and support to athletes on healthy eating and access to specialists who can help identify and manage eating problems.

## Figures and Tables

**Table 1:** Level of football competition in Poland [18].

Level	Name Od Competition Level	Status of the Competition	Sports Level
1	Ekstraklasa	Central games	Professional football
2	I league	Central games	Professional football
3	II league	Central games	Professional football
4	III league	Central games	Professional football
5	IV league	Regional games	Amateur football
6	District class	Regional games	Amateur football
7	A class	Regional games	Amateur football
8	B class	Disctrict games	Amateur football
9	C class	Disctrict games	Amateur football

**Table 2:** BMI classification according to WHO [19].

BMI (kg/m^2^)	Interpretation of BMI
<18.5	Underweight
18.50–24.99	Body weight normal
25.00–29.99	Overweight
30.00–34.99	First-degree obesity
35.00–39.99	Second-degree obesity
≥40.00	Third-degree obesity

**Table 3:** Interpretation of men’s BMI compared with norms for age [19].

Age	9	10	11	12	13	14	15	16	17	18	19	20	>20
BMI-male	14.0	14.5	15.0	15.0	16.0	16.5	17.0	17.5	18.0	18.5	19.0	19.5	20.5

**Table 4:** Age-specific interpretation of BES for men [23].

Stens	16–19 Years			20–29 Years			30–39 Years		
	PA	UBS	PC	PA	UBS	PC	PA	UBS	PC
1	≤26	≤20	≤29	≤28	≤23	≤32	≤28	≤22	≤32
2	27–30	21–23	30–34	29–31	24–26	33–36	29–31	23–25	33–36
3	31–33	24–26	35–38	32–34	27–29	37–40	32–34	26–28	37–40
4	34–36	27–30	39–43	35–37	30–31	41–45	35–37	29–31	41–44
5	37–39	31–33	44–47	38–41	32–34	46–49	38–40	32–34	45–48
6	40–43	34–36	48–52	42–44	35–37	50–53	41–44	35–36	49–52
7	44–46	37–39	53–56	45–47	38–40	54–57	45–47	37–39	53–56
8	47–49	40–42	57–61	48–50	41–43	58–61	48–50	40–42	57–60
9	50–52	43–45	62–65	51–54	44–46	62–65	51–53	43–45	61–64
10	≥53	≥46	≥66	≥55	≥47	≥66	≥54	≥46	≥65

PA—Physical Attractiveness; UBS—Upper Body Strength; PC—Physical Condition.

**Table 5:** Characteristics of the study group (n = 90).

	Total (n = 90)	AF (n = 59)	PF (n = 31)	*p*-Value
Age [years] (X ± SD)	28.21 ± 5.11	28.52 ± 4.48	27.65 ± 6.18	0.450
Height [cm] (X ± SD)	181.41 ± 6.62	179.1 ± 5.86	185. 81 ± 5.75	0.001 *
Body mass [kg] (X ± SD)	78.92 ± 8.36	77.62 ± 8.37	81.42 ± 7.89	0.04 *
BMI [kg/m^2^] (X ± SD)	23.95 ± 1.91	24.15 ± 1.9	23.57 ± 1.89	0.171
Sports seniority [years] (X ± SD)	18.82 ± 4.77	19.58 ± 4.65	17.38 ± 4.74	0.038 *
Training units per week (X ± SD)	3.48 ± 1.3	3.5 ± 0.51	4.77 ± 1.02	0.001 *

AF—amateur footballer; PF—professional footballer; *—*p* < 0.05; X—average; SD—standard deviation.

**Table 6:** Exclusions of products from the diet and how to adapt the diet to increased physical activity declared by athletes (n = 90).

Sports Level	AF (n = 59) n (%)	PF (n = 31) n (%)	Total (n = 90) n (%)	*p*-Value
Exclusions of food products from the diet				
I do not exclude	39 (66.10)	19 (61.29)	58 (64.44)	0.579
Red meat	2 (3.39)	1 (3.23)	3 (3.33)	
Fruits	2 (3.39)	0	2 (2.22)	
Fish and seafood	2 (3.39)	6 (19.35)	4 (4.44)	
Nuts	2 (3.39)	1 (3.23)	3 (3.33)	
Monosaccharides	2 (3.39)	0	2 (2.22)	
Products contain lactose	10 (25.64)	6 (19.35)	16 (17.78)	
How to adapt diet to increased physical activity				
Increase carbohydrate intake	3 (5.1)	14 (45.2)	17 (18.9)	0.001 *
Increase fluid intake	25 (42.4)	13 (41.9)	38 (42.2)	0.969
Increase protein intake	17 (29.3)	18 (51.4)	35 (39.3)	0.006 *
Reduce fat intake	6 (10.2)	1 (3.2)	7 (7.8)	0.247
Restrict the consumption of sweets	14 (23.7)	0	14 (15.6)	0.003 *
Increase the energy intake on training/match days	9 (15.3)	14 (45.2)	23 (25.6)	0.002 *
Eating before and after physical activity	12 (20.3)	11 (35.5)	23 (25.6)	0.120 *

AF—amateur footballer; PF—professional footballer; *—*p* < 0.05.

**Table 7:** Football players’ social media activity by sports level (n = 90).

Sports Level	AF (n = 59)	PF (n = 31)	Total (n = 90)	*p*-Value
Time of use of social media during the day n (%)				
Up to 1 h	15 (25.4)	0	15 (16.7)	0.009 *
1–2 h	21 (35.6)	18 (58.1)	39 (43.3)	
2–3 h	15 (25.4)	6 (19.4)	21 (23.3)	
above 3 h	8 (22.6)	7 (16.7)	15 (16.7)	
The most common type of social media n (%)				
Tik-Tok	6 (10.2)	5 (16.1)	11 (12.2)	0.086
Instagram	21 (35.6)	17. (54.8)	38 (42.2)	
Twitter	5 (8.5)	4 (12.9)	9 (10)	
Facebook	24 (40.7)	5 (16.1)	29 (32.2)	
Different	3 (5.1)	0	3 (3.3)	
Purpose of using social media n (%)				
Relax	44 (74.6)	25 (80.6)	69 (76.7)	0.523
I’m looking for information on sports	29 (49.2)	16 (51.6)	45 (50.0)	0.827
I look for information on diet/nutrition	16 (27.1)	11 (35.5)	27 (30.0)	0.416
I look for the news of the day	35 (59.3)	21 (67.7)	56 (62.2)	0.439
I check what’s going on with friends	33 (55.9)	27 (87.1)	60 (66.7)	0.003 *
Comparing body image to photos of other players on social media n (%)				
no, never	41 (69.5)	13 (41.9)	54 (60)	0.023 *
yes, sometimes	12 (20.3)	14 (45.16)	26 (28.9)	
yes, often	6 (10.2)	4 (12.9)	10 (11.1)	

AF—amateur footballer; PF—professional footballer; *—*p* < 0.05.

**Table 8:** Summary of ED risk estimation (EAT-26) (n = 90).

EAT-26	Total		AF (n = 59)		PF (n = 31)		*p*-Value
Elevated Risk	No Risk	Elevated Risk	No Risk	Elevated Risk	No Risk	Elevated Risk	
Part A (X ± SD)	82 (91.1)	8 (8.9)	52 (88.1)	7 (11.9)	30 (96.8)	1 (3.2)	0.171
Part B (X ± SD)	81 (90.0)	9 (10.0)	53 (89.8)	6 (10.2)	28 (90.3)	3 (9.7)	0.941
Part C (X ± SD)	87 (96.7)	3 (3.3)	57 (96.6)	2 (3.4)	30 (96.8)	1 (3.2)	0.967
Entire (X ± SD)	75 (83.3)	15 (16.7)	48 (81.4)	11 (18.6)	27 (87.1)	4 (12.9)	0.487

AF—amateur footballer; PF—professional footballer.

**Table 9:** Assessment of the athletes’ body attractiveness according to the number of sten and BES interpretation (n = 90).

	Total (n = 90)			AF (n = 59)			PF (n = 31)			*p*-Value
PA [sten] X ± SD	5.80 ± 2.27			5.31 ± 2.33			6.74 ± 1.86			0.008 *
UBC [sten] X ± SD	5.63 ± 2.22			5.29 ± 2.17			6.29 ± 2.21			0.031 *
PC [sten] X ± SD	5.79 ± 2.33			5.41 ± 2.35			6.52 ± 2.16			0.039 *
**Assessment of the Attractiveness Subscale:**	**Low**	**Medium**	**High**	**Low**	**Medium**	**High**	**Low**	**Medium**	**High**	***p*** **-Value**
PA n (%)	22 (24.4)	47 (52.2%)	21 (23.3)	20 (33.9)	27 (45.8)	12 (20.03)	2 (6.5)	20 (64.5)	9 (29.0)	0.016 *
UBS n (%)	22 (24.4)	47 (52.2%)	21 (23.3)	17 (28.8)	33 (55.9)	9 (15.3)	6 (16.1)	14 (45.2)	12 (38.7)	0.037
PC n (%)	20 (22.2)	50 (55.6)	20 (22.2)	16 (27.1)	36 (61.0(	7 (11.9)	4 (12.9)	14 (45.2)	14 (41.9)	0.004 *

PA—Physical Attractiveness; UBS—Upper Body Strength; PC—Physical Condition; AF—amateur footballer; PF—professional footballer; *—*p* < 0.05.

**Table 10:** Relationship between EAT-26 score and BES.

EAT-26	Low	Average	High	*p*-Value	V Cramer
	PA				
**No risk**	15 (20.0)	41 (54.7)	19 (25.3)	*p* = 0.085	0.234
**Elevated Risk**	7 (46.7)	6 (40.0)	2 (13.3)		
	UBS				
**No risk**	14 (18.7)	43 (57.3)	18 (24.0)	*p* = 0.014 *	0.307
**Elevated Risk**	8 (53.3)	4 (26.7)	3 (20.0)		
	PC				
**No risk**	12 (16)	46 (61.3)	17 (22.7)	*p* = 0.005 *	0.343
**Elevated Risk**	8 (54.3)	5 (26.7)	3 (20.0)		

PA—Physical Attractiveness; UBS—Upper Body Strength; PC—Physical Condition; *—*p* < 0.05.

## Data Availability

The raw data supporting the conclusions of this article will be made available by the authors upon request.
